# Expanding options to recruit, grow, and retain the public health workforce

**DOI:** 10.1093/haschl/qxae115

**Published:** 2024-12-04

**Authors:** Kate Beatty, Laura Hunt Trull, Christen Minnick, Kawther Al Ksir, Kristen Surles, Michael Meit

**Affiliations:** Department of Health Services Management and Policy, College of Public Health, East Tennessee State University, PO Box 70264, Johnson City, TN 37614, United States; Center for Rural Health Research, College of Public Health, East Tennessee State University, PO Box 70264, Johnson City, TN 37614, United States; Department of Health Services Management and Policy, College of Public Health, East Tennessee State University, PO Box 70264, Johnson City, TN 37614, United States; Center for Rural Health Research, College of Public Health, East Tennessee State University, PO Box 70264, Johnson City, TN 37614, United States; Center for Rural Health Research, College of Public Health, East Tennessee State University, PO Box 70264, Johnson City, TN 37614, United States; Department Community and Behavioral Health, College of Public Health, East Tennessee State University, PO Box 70264, Johnson City, TN 37614, United States; Center for Rural Health Research, College of Public Health, East Tennessee State University, PO Box 70264, Johnson City, TN 37614, United States; Department Community and Behavioral Health, College of Public Health, East Tennessee State University, PO Box 70264, Johnson City, TN 37614, United States; Department of Health Services Management and Policy, College of Public Health, East Tennessee State University, PO Box 70264, Johnson City, TN 37614, United States; Department of Health Services Management and Policy, College of Public Health, East Tennessee State University, PO Box 70264, Johnson City, TN 37614, United States; Center for Rural Health Research, College of Public Health, East Tennessee State University, PO Box 70264, Johnson City, TN 37614, United States

**Keywords:** career ladders, public health workforce, retention

## Abstract

The public health workforce continues to atrophy due to mass and early retirement, under-funding, slow hiring processes, lack of advancement opportunities, and shifting policies. Organizational research into workforce sustainability is crucial for ensuring a robust, diverse staff capable of delivering essential public health services. We examined career ladders, a potential solution to workforce challenges, through interviews with 10 health departments (HDs) across seven states. Interview participants were recruited from HDs using or planning career ladders held administrative positions, and had a role in the hiring process.

Many health department positions have traditionally included steps within certain job classifications that promote pay adjustments with increasing years of service. Career ladder approaches, however, specifically focus on providing opportunities for health continuing education, leadership development, or movement into formal leadership roles.

Findings indicate that HDs have begun utilizing career ladders for professional development and critical role maintenance. Career ladders have been applied mostly for retention with limited impact on recruitment and increasing staff diversity. Challenges include civil service requirements, funding limitations, and complex recruitment that might exclude diverse candidates.

This study emphasizes the importance of transparent development, engaging front-line staff, offering advancement pathways, and providing insights to enhance workforce recruitment and retention.

## Teaser

The public health workforce plays a critical role in ensuring positive health outcomes for patients; however, workforce atrophy is a problem the field faces. Lack of viable advancement opportunities limits both recruitment and retention opportunities in public health systems. This study examined career ladders as a potential solution for recruitment and retention issues within public health agencies. Findings emphasize the challenges facing the public health workforce as well as opportunities for mitigation strategies in public health settings.

## Introduction

The governmental public health workforce has traditionally faced a variety of recruitment and retention challenges that impact its ability to maintain a robust and diverse staff capable of delivering essential public health services to communities.^1-3^ An aging workforce, higher turnover rates, lower compensation, slower hiring processes, and decreases in public funding compound public health leaders’ ability to recruit, grow, and retain a competent workforce.^1,4-6^ While the COVID-19 response certainly impacted local health department staffing levels, recent PH WINS (Public Health Workforce Interests and Needs Survey) analyses indicate that trends in public health workers considering leaving their agencies predate the pandemic. These personnel management burdens have required public health leaders to identify new strategies to engage and retain competent employees. While more commonly used in private sector organizations, career ladders may provide an opportunity for governmental public health agencies to improve their ability to recruit and retain their workforce by demonstrative long-term career growth opportunities.^2,3,5,7-9^

### Career ladders

Career ladders are mechanisms that provide workers with a formal path, like education, certification, and performance requirements, to follow to advance to the next level within their respective fields and careers. Career ladders may provide an opportunity for entry-level employees to gain knowledge and skills needed to advance to intermediary and leadership positions within their agency.^7,10,11^ Career ladders have a series of official steps within a job classification which increase in responsibility and required expertise with each step.^12^ Career ladders build on the existing job classifications to establish a formal professional development path for workers to intentionally move to the next level of the ladder, such as education, certification, and performance requirements. Often, career ladder programs partner with local higher education institutions like colleges, universities, and training organizations to provide the necessary education for employees to advance.^10,11^ Rather than unpredictable moves through job steps or stalling at certain points in a vocation, career ladders help agencies recruit, grow, and retain knowledgeable staff and improve employees’ wages and job satisfaction.^7,10,11^

Increasingly, many industries lack the positions needed to bridge the gap between entry-level and low-wage positions and leadership positions.^10^ As such, the development and utilization of career ladder programs require organizations to restructure or create new positions with advancement potential. However, more rigid hierarchies, burdensome hiring and job development processes, and challenges in recruiting entry-level employees with formal public health education may limit governmental public health agencies’ flexibility in bridging this gap.^1,7,11^ While career ladders can improve employee satisfaction and wages, as well as agencies’ ability to recruit and retain skilled workers, little is known about if and how governmental public health agencies implement career ladders, nor how they may impact their ability to recruit and retain a diverse workforce.

Like career ladders, succession planning offers opportunities for workers to advance within their organizations. Succession planning is a way for organizations to identify critical leadership and management positions and develop staff to assume those positions.^2,3,5,8,9^ Through succession planning, organizations can ensure continuous leadership, preserve institutional knowledge, and improve their ability to recruit and retain a skilled workforce. As such, succession planning is essential for the sustainability of an organization.^13^

### Diversity

As the United States population becomes more diverse, the need for a more diverse public health workforce becomes more evident.^1,14,15^ Currently, 40% of American adults and over 50% of youth under the age of 16 identify as a racial or ethnic minority.^1,15^ While an average of 42% of governmental public health workers identify as a racial or ethnic minority,^15^ representation of people of color varies widely by region and is lower among some public health professions, and among public health leaders.

Data from the 2017 PH WINS show that most persons of color within public health agencies work in administrative or clerical positions at state and local health departments.^15^ On the other hand, public health science positions were mostly occupied by non-Hispanic Whites in state and big city health departments. Overall, a greater proportion of non-Hispanic Whites held leadership positions compared to minority groups.

Recruiting, growing, and retaining a diverse public health workforce better equips governmental public health agencies to implement more directed and effective solutions to address their communities’ needs.^15^ A more diverse workforce that better reflects the communities served, enhances public health agencies’ ability to provide culturally and linguistically appropriate services. While research suggests that the use of career ladders or succession planning programs may help improve the diversity within public health agencies, little is known about how these tools are used to recruit and retain a diverse public health workforce. To enhance this understanding, this study examined how public health leaders define and utilize career ladders and facilitators and barriers to their use as well as identify strategies to strengthen the use of career ladders in governmental public health.

## Methods

### Data sources

The 2019 National Profile of Local Health Departments data from the National Association of County and City Health Officials (NACCHO) was used to describe the organizational and community characteristics of local health departments that were selected and interviewed.^16^ Additionally, a few state-level agencies also participated in the interviews. Data on organizational and community characteristics of state health departments was pulled from the Association of State and Territorial Health Officials (ASTHO) Profile of State and Territorial Public Health (https://www.astho.org/topic/public-health-infrastructure/profile/).^17^

### Selection of health departments

This study used health department workforce development plans (WDPs)submitted to the Public Health Accreditation Board (PHAB) to identify health departments (HDs) that use or were planning to use career ladders or succession planning. WDPs are part of the accreditation process and provide information on agency-level workforce gaps and the plans and strategies that HDs are implementing or plan to implement to address those gaps. These two terms were selected because while there are fine distinctions, people tend to use these and other terms interchangeably to mean “career ladders.”^18^ PHAB requires HDs to provide a WDP from the past five years articulating specific objectives and strategies they plan to undertake to achieve its desired future workforce, which could include career ladders and/or succession planning as a strategy.

To qualitatively assess WDPs submitted to the PHAB, existing data from the CWORPH (Consortium for Workforce Researchers in Public Health) members’ data use agreement with PHAB were utilized. The WDPs were collected from PHAB between November 2021 and January 2022 and included state and local HDs that were accredited or reaccredited between March 2016 and November 2021. The sample included 168 initial accreditation plans and 33 reaccreditation plans. A team of 11 coders reviewed and extracted information from the WDPs that included information related to career ladders and succession planning. Details on this methodology can be found elsewhere.^19^

Thirty-nine health departments identified and described career ladders and/or succession planning as one of the following (1) a gap in their WDP, (2) a strategy that they included in their plan, or (3) that they intend to explore career ladders or succession planning in the future. Three team members reviewed the WDPs for each identified health department, pulling out all language related to career ladders and succession planning. The team coded the content and identified health departments that were either using career ladders or succession planning or intended to do so. As the WDPs are a point-in-time document, they capture health departments that were planning or very new to career ladders, as well as those who had integrated career ladders into their processes for some time. These health departments (*N* = 26) were contacted and invited to participate in an interview to learn more about how their health departments may be utilizing career ladders and/or succession planning, as well as successes and challenges with career ladders and/or succession planning. Additionally, discussions explored how they might improve workforce recruitment, retention, and diversity. PHAB facilitated the connection between the health departments and the researchers. Participants from health departments were contacted via email by PHAB, introducing the study and the researchers and inviting health departments to participate. PHAB reached out to each health department up to three times. Researchers were given health department contact information to follow up if they agreed to participate. Further correspondence occurred solely between the researchers and the health departments.

### Health department interviews

A semi-structured interview guide was developed to gain insights into how career ladder programs were implemented within the organizational context of public health agencies. Individuals with knowledge of the use of career ladders were identified by health departments and invited to participate in interviews. These semi-structured interviews were conducted via Zoom with researchers. Participants provided verbal consent at the outset of the interview, including consenting for recording. Interviews averaged about 45 minutes (range of 30-65 minutes). Interviews were recorded and transcribed. This study was reviewed and approved by the Institutional Review Board of East Tennessee State University.

### Data analysis and coding

Coding was completed in three phases. During the first phase, a primary coder and two secondary coders used a rapid thematic approach to develop a summary matrix of interview responses and identify emergent themes.^20,21^ During the second phase, interrater agreement between primary and secondary coders was analyzed. Differences were identified and resolved using consensus-based discussions. The template was subsequently used to summarize the remaining transcripts by one of those researchers. Lastly, emerging themes were highlighted and agreed upon by the coding team.^20-22^

## Results

### Respondent characteristics

Semi-structured key informant interviews were conducted from June 2023 to August 2023 with health department representatives who were identified as using or planning to use career ladders and/or succession planning as part of their workforce development plans. Participants’ experience with career ladders varied from very new to seasoned integration in their professional development plans. Interviews were conducted with 14 participants representing 10 health departments across seven states. Most of the interviews were conducted with county or city/county health department personnel (*n* = 7), while three interviews were with state health department personnel. Health departments represented all four Census regions of the US and were representative of all major governance structures. Health department staff ranged in size from 19 to 3652 full-time equivalents who served populations from 14 406 (Midwestern county health department in a decentralized state) to 10.8 million (Southern state health department with a decentralized structure [see Table 1]). All the participants were at the director, deputy director, supervisor, manager, or coordinator level and had a role in the hiring process as well as supervising, promotion, and retention efforts within their department. Some were responsible for hiring at the state level, and some state respondents were responsible for informing policies that local health departments utilize to fill vacancies. Local health department respondents were either responsible for hiring their direct reports or for signing off on the hiring process conducted by supervisor or manager-level staff. Interviewees discussed recruiting, hiring, and retaining a diverse workforce, how career ladders are defined and utilized, and facilitators and barriers to implementing career ladders in their health department, as well as recommendations for their improvement.

**Table 1.** Organizational characteristics of participating health departments (*N* = 10).

Health department	Jurisdiction	Region	Governance structure	FTE	Population size served
1	County	West	Decentralized	93	188 987
2	State	Midwest	Decentralized	419	5.896 million
3	State	South	Centralized	3652	5.191 million
4	County	Midwest	Decentralized	28	14 406
5	State	South	Shared	986	10.8 million
6	County	South	Shared	440	31 659
7	County	Midwest	Decentralized	—	109 264
8	County	Northeast	Mixed	368	1.223 million
9	County	West	Decentralized	86	180 930
10	City/County	Midwest	Decentralized	19	66 597

Abbreviation: FTE, Fulltime Equivalent Employee.

### Recruiting and retaining health department staff

To provide context around hiring and retaining staff, health departments reported extensive staffing challenges during and following the COVID-19 pandemic. Challenges included turnover and burnout, an inability to hire or compensate adequately, and difficulties filling open positions, especially specialized positions. Many health departments are required to follow state or local policies regarding advertising a position and screening applicants. Despite governance structure, state-level Civil Service requirements may still impact the hiring process, even if a local team conducts interviews and makes hiring recommendations, which can create challenges for the implementation of career ladders and formal succession planning.

When discussing efforts to increase equity and diversity in the hiring processes, some health departments described innovations in developing new tools to make applying for jobs easier for a wider pool of applicants. Additional recruitment strategies included updating position descriptions to highlight lived experiences. More broadly, health departments were partnering with higher education institutions including community colleges and programs, schools, and colleges of public health for interns to create career pipelines and support movement up career ladders.

Health department respondents also identified a few strategies for retaining and growing their staff. They all reported challenges with salary and compensation, with some actively addressing this through salary adjustments. Additionally, respondents discussed becoming more flexible by supporting more telecommuting and moving to 10-hour workdays. These flexibilities have become more important to staff since the pandemic, especially with compensation constraints. Importantly, career ladders were identified as a key strategy for retention at health departments.

### Operationalizing career ladders

Respondents described career ladders as mostly internal processes for current employees– to improve staff positions and salaries through increased education or responsibility. “*[W]e recognize that we have lost very good employees just because they recognize what the ceiling is here. At this time we don’t have a lot of turnover in our leadership rows either. So, we recognize the need to put employees in positions where they could have additional responsibilities and develop those skills”.* For some, this was a formal process with clear guidelines on advancement up the rungs of the ladder.

Several individuals reported that career ladders are embedded in professional development plans for current staff. Respondents stated that staff know what additional experience, education, or training they need to move to the next step. This was operationalized as a series of standardized steps within roles, titles, and salary increases, such as Nutritionist I to Nutritionist II to Nutritionist III to Nutrition Supervisor. In some cases, these were linked to succession planning as they were used to plan for supervisor departure with a preference for internal candidates. Among some health departments, the career ladder was automatic, and staff could move up a step in salary and/or responsibility based on the number of years in service, while other health departments required staff to take initiative in their advancement. Another example of clinical staff is nursing moving from a licensed practical nurse I (LPN I) to an LPN II and with educational attainment to a registered nursing (RN) classification. With training and leadership development, a nurse could move into a supervisor role. Non-clinical examples include health educators and epidemiologist staff who can move into higher classifications with training and experience. Often job classifications already exist with both automatic steps based on experience and planning to support professional development for staff to move up the ladder. In a few cases, respondents discussed developing more “rungs” to support movement up a career ladder.

### Effectiveness of career ladders

Career ladders were described as an effective tool for retaining employees. Several participants noted that they have a career ladder established for one or two positions and are looking to expand to have them for numerous or all positions. Respondents found that career ladders were linked to improved employee satisfaction. For example, “*I can tell you people are more engaged. We’ve got more discussions going on in all staff meetings about careers and career ladders. We’ve got some people really grateful and appreciative because a lot of our staff really care about the communities and the vulnerable populations that we serve*” (Table 2).

**Table 2.** Representative quotes for health department representatives.

Representative quotes	
**Career Ladder Operationalization**	
Tool for individual development planning	We deem you to have this much experience, and so, therefore, you will get that pay. What we didn’t do is do anything more than that year after year. So anyways, next year we will evaluate at their annual review period if they’ve moved into a new category of years experience, they’ll move up in salary (state health department, centralized state)
Development beyond advancement	So, we recognize the need to put employees in positions where they could have additional responsibilities and develop those skills (local health department, micropolitan area in a decentralized state)
Requires individual initiative	“Well, if I’m going to move up in this business, what’s it going to take?” A person has to take the initiative (local health department, rural nonmetropolitan area in a shared governance state)
Through succession planning	Also, we were making succession plans. Each manager had a succession plan in mind with contingencies, obviously, to make sure that the folks were able to continue to rise within the organization (local health department, metropolitan area in a mixed structure state)
Incentives for higher education	So, you move from 0 to 2 years experience, and you move into 3 to 5 category experience and you get a thousand dollars. It would be nice for it to be larger. It would also be nice to have additional educational attainment rewards in there, as well. We don’t have that yet (state health department in a centralized state)
Clear guidance on advancement up rungs	I think it would be nice to be able to give people stepping stones? I can’t think of the word I’m trying to think of, but you know what I’m saying. Little steps along the way to get there, so that you have small increments of increases or some sort of benefit along the way (regional health department in a shared governance state)
**Effectiveness of Career Ladders**	
Staff satisfaction & retention	I can tell you people are more engaged. We’ve got more discussions going on in all staff meetings about careers and career ladders. We’ve got some people really grateful and appreciative because a lot of our staff really care about the communities and the vulnerable populations that we serve (local health department, metropolitan area in a decentralized state)
**Importance to Longevity and Retaining Institutional Knowledge**	
Prepares staff more high positions	It’s investing in the staff, and they spend true time, and I think they get a lot out of it from the organization. The organization allows me to promote, they want me to promote, they’re giving me space, and it’s more than just the career ladder (local health department, metropolitan area of a decentralized state)
Little or no immediate impact	I don’t know that it necessarily made a change now. Well, I mean, it’s helping us plan and prepare, but we’re not really seeing effects, I guess, yet (local health department, metropolitan area in a decentralized state)
More transparency in hiring process	I think it would just make it a little clearer for people to know what to expect. I think they’d be more settled, long term, of their path forward.” (state health department, decentralized state)
Improved culture	We just funded one of our community health advocates to take a course down at [university] in medical interpretation because she’s English as a second language, and she’s thrilled. She’s so excited to put what she’s going to learn into practice because it’s about the folks that she works with that are literally her neighbors. She’s looking forward to being able to do a better job and have more tools in her tool belt. I think those are the stories that resonate the most with me in terms of we’re not only changing the lives of our workforce but, in turn, reaching that deeper into the community and making a bigger impact in the community where we really try to focus and serve (local health department, micropolitan area in a decentralized state)
Promotes more collaboration	Yeah, you have to insist that we do the job, and you show up for work, and that you do quality work. But you have to build the camaraderie and the teamwork and the ability to work together. …And you need to rely on and trust and enjoy the people you work with (local health department, rural nonmetro area in a decentralized state)
**Challenges with Career Ladders**	
Regulatory and hiring misalignment	Because of the way that we operate, even if I say “Jane” is my successor, I can’t actually make “Jane” my successor because we have to operate under Civil Service. The way that civil service works you have to post the job, rate all the candidates, and things like that. So if I say “Jane” as my successor and then I leave, and they don’t hire “Jane” then that doesn’t work out (local health department, metropolitan area in a mixed structure state)
Limited growth paths	A lot of people have complained or left because the jobs that they’ve accepted go nowhere. There is no career ladder. You get one or two steps, and then you’re at that fixed rate forever. And so people don’t want to stick around if the only thing they’re going to get is a 1.5 or 2% cost of living increase every four years it it’s a dead end. So we’re trying to find these dead ends and build bridges to other ladders, and that’s the workforce development side (local health department, metropolitan area in a decentralized state)
**Specialized role fulfillment**	
Inefficiencies in the hiring process	The recruitment process's complexity leads to losing potential candidates to other job offers, while internal movement within the organization can also lead to turnover. Though money can address some issues, it is not a sole solution, and a study on hiring procedures could prompt organizations to streamline and improve the process (local health department, micropolitan area in a decentralized state)
Hard-to-fill roles	And then the other piece is that there have been historically difficult-to-fill positions that are even more difficult to fill. So, things like microbiologists, physical therapists, and public health nurses. Certainly, even harder to fill would be the leadership in those positions.” (local health department, metropolitan area of a decentralized state)
**Financial and promotional balance​**	
Public v/s private pay gaps	Career paths are great if you can compete with the private sector. But funding wise, our pay grade levels are lower than what you would get out in the private sector. So that’s probably the challenge that we have is funding (local health department in a micropolitan area of a shared governance state)
Financial resources for increases	We have to come up with the money locally, which, of course, puts some health departments where we may not be able to hire as many nurses or as many environmentalists, because of having to cover the higher salaries. All of our salary administration itself is covered by the State Personnel Board rules in regard to promotions and things like that within the career ladder (local health department in a micropolitan area of a shared governance state)
**Policies Impacting Career Ladders**	
Hiring policies hinder	The place that I think we have a little bit more limitations is our interview process. It is in state statute and then by our different state agency’s interpretation of how that state statute applies of what we are able to do as far as number of candidates, what we can have them do, transparency of questions, which I think, gets back to the DEI pieces as well (state health department in a decentralized state)
Educational and training requirements	I do think some of the education requirements are too for positions. So, it’s hard enough to get environmental health specialists. And then, through the state, you have to show certain classes through a 4-year degree. And there are a lot of hoops to jump through to get that environmental health and training certificate to practice (local health department, metropolitan area in a decentralized state)
BOH flexibility seen as a benefit	I’m very fortunate to have an extremely Supportive Board of Health who recognizes that we’re doing good work. They give us the feedback we need on how to serve our community, but they do not micromanage (local health department, rural nonmetro area in a decentralized state)
**Improving Career Ladders**	
Transparency	So, I think for me, the takeaway is when we’re looking at any of these interventions or policies and strategies for workforce recruitment and retention, that we need to remember it’s all interconnected. I tend to say that workforce needs to start with the culture first (state health department, decentralized state)
Competitive and innovative measures	[W]e really sat down and were thoughtful about the structure of our programs and making sure that there were those intermittent steps and you weren’t just down here with the goal to be up here and no steps in between. We’ve really done a good job across the department of being thoughtful as we’re reconfiguring our organizational charts and how to make sure that folks have stepping stones to get to the leadership here in the health department (local health department, metropolitan area in a mixed structure state)
Tailoring ladders for professional growth	I would like to toot the horn on advice for our individual development program boot camps. [O]ur leadership has provided the okay for [organization] to build these spaces on company time for individuals to spend, and you know it’s several sessions, so it’s not just a come to a working lunch and let us tell you about great work opportunities. It’s investing in the staff, and they spend true time, and I think they get a lot out of it from the organization. Like oh, the organization allows me to promote, they want me to promote, they’re giving me space, and it’s more than just the career ladder… (local health department, metropolitan area of a decentralized state)
Incremental leadership responsibilities	“[W]e recognize the need to put employees in positions where they could have additional responsibilities and develop those skills. And even if that means that they’re developing skills to take somewhere else, that’s okay with us. Because it’s not just about, yeah, do we want to keep the best? But we want to treat people well also. And if that means that they have the leadership skills and have developed skills to take somewhere else. Well, they’re just taking their public health mindset into another organization in the community, which will only help the greater goal of the public health of our community.” (local health department, micropolitan area in a decentralized state)
Cultivate flexibility and adaptability	So, I think we need to apply the career ladder idea and principle of that investment, but then have that flexibility and adaptability to what we are experiencing so people like myself and others can see the leadership skills, see the growth in a different way, and create some ways that both from a salary standpoint, benefits standpoint, and an investment standpoint that we’re growing with people in the trajectory of their career (state health department, decentralized state)
**Career Ladders and Diversity**	
No impact on DEI	I would say we work really hard to try to recruit among and from and within diverse groups. We wanna look like our communities. But I wouldn’t say that the career ladder… The career ladder helps us to be fair to everyone. But I wouldn’t say that specifically helps us become more diverse. I think that would be a stretch if we’re gonna say that (state health department in a centralized state)
Staff should reflect community	I feel that we try. We don’t always get a diverse applicant pool. You know, we try to branch out every way we possibly can. But I also can say in our report back from PHAB when we submitted this. They did say that we could do better.” (local health department, metropolitan area in a decentralized state)
Enhance recruitment	Now broadening our scope to LinkedIn and Indeed and other platforms and having the recruiter mechanism involved in LinkedIn, we’re able to get pretty diverse candidates, which we’ve been happy with. As far as retention on the career ladder of more diverse candidates, I don’t know that we’ve actually done an analysis to figure out whether that’s been the trend or not. [T]he retention numbers for candidates that we’re recruiting are increasing because of the way that we’re proceeding with these (local health department, metropolitan area in a mixed structure state)
DEI and long-term goals	Maybe they’re a person that answers the phone at the front desk and they’re bilingual, so they’re able to communicate, but what they really want to do is to be a nurse or work in epidemiology. How can we help them get there? Our typical response is to advertise for those positions rather than the hard work which is filled from within. Support those individuals with work–life balances, support them with career-building opportunities, financial support, and build those people with within. Because they want to live and work in their community. They want to help their neighbors. That is your biggest strength. Why are we just putting ads in the paper out there and ads online because all we care about is filling the position. But how successful is that? (local health department, metropolitan area in a decentralized state)

Abbreviations: DEI, diversity, equity, and inclusion; BOH, Board of Health.

Effective career ladders were reported to recognize longevity with the health department and education/training advancement. The use of career ladders was seen as preparing staff for higher positions within the organization, supporting the goals of retention and succession planning. These efforts also improved the culture of the health department by helping staff feel invested in health department goals and objectives within the community. Additionally, health department leadership reported that when there were funds to support educational opportunities for current staff, they were more likely to move up the career ladder.

Some health department respondents stated that their departments were early in their implementation of career ladders and have seen little or no impact of career ladders at this time. One respondent stated, “*I don’t know that it necessarily made a change now. Well, I mean, it's helping us plan and prepare, but we’re not really seeing effects, I guess, yet”* (Table 2).

### Challenges in the use of career ladders

The most common barrier to implementing career ladders was the funding associated with the salary increases to move up a ladder. Health department respondents reported being reliant on state leadership to make budget decisions and allocate funds, which at times were inadequate. Respondents also reported hesitancy in fully implementing formal career ladders as it would require higher salaries and reclassifications at each level of advancement, which would be contingent on funding.

Formal career ladders were limited often to only a few positions and there were limited growth paths, “*[W]e recognize that we have lost very good employees just because they recognize what the ceiling is here. At the time we don’t have a lot of turnover in our leadership roles either*” (local health department, micropolitan area in a decentralized state). Respondents also described that career ladders can be rigid and prescriptive, making it a challenge for staff to realize potential opportunities. Career ladders were not thought to be useful in recruitment, and progress through the career ladders could be negatively impacted by inefficiencies in the hiring process which contribute to gaps and workforce disruptions. Additionally, health department respondents discussed regulatory and hiring misalignment where there were differences between hiring desires and civil services regulations. Leaders expressed frustration that a current employee could be training for a position and lose out to an unaffiliated candidate who happens to look better on paper. The policies that regulate hiring were thought to be a barrier as most health departments are required to promote positions openly and publicly. Participants also identified a ripple effect in following hiring policies that impact their general ability to fill vacancies. They described that these policies may impact departmental goals related to diversity, for example, by requiring positions to be advertised and applicants to be blind screened.

### Strategies

Multiple themes emerged to improve the use of career ladders in health departments. One of the larger strategies discussed was to reconfigure organizational structures through organizational charts and job classifications to include more stepping stones for advancement, adding rungs to the career ladder for smoother progression. This also included creating career development plans for positions. In many instances, health department leaders indicated that career ladders that are developed in partnership with staff promote transparency in the process. By engaging with front-line staff in the development and utilization of career ladders, they noted that leadership clearly communicates the education and training requirements needed to meet professional development goals and be flexible in meeting the changing needs of the workforce. They described that this can help maintain a competent workforce by limiting the impact of attrition due to retirement, burnout, or individuals leaving for better-paying opportunities.

Some strategies that can be implemented on a smaller scale were also identified. One was to tailor ladders for professional growth by creating customized career ladders for staff and addressing educational requirements through smaller, more incremental steps. Respondents also discussed the idea of incremental leadership responsibility by providing staff without formal leadership roles with more leadership duties to foster skill development and career progression. Finally, in addition to more rungs and leadership opportunities, respondents recommended the adoption of innovative policies like remote work, to attract and retain staff.

## Discussion

The findings from the interviews with representatives from 10 health departments across seven states shed light on the emerging significance of career ladders in public health workforce administration, particularly in response to the challenges presented by the post-COVID-19 era. Recently, local health departments have grappled with recruitment and retention obstacles due to lower wages and a limited pool of applicants possessing the required specialized training for essential public health roles.^23^ The additional strain imposed by the pandemic has intensified these difficulties, prompting the adoption of innovative strategies to address employee stress, burnout, and pay dissatisfaction.^24^ Approaches like career ladder implementation and succession planning are traditional private sector tools that health departments can tailor to meet their unique operational needs. This shift in approach for personnel management has been supported by a national post-pandemic focus on cultivating a proficient public health workforce, with increased funding that may facilitate wage adjustments and support new recruitment and retention activities.^25,26^

Overall, respondents described that career ladders play a key role in retaining existing staff by providing a clear path for professional growth, at times leading employees to progress from entry-level positions to leadership roles. However, their impact on recruitment efforts and fostering diversity is less pronounced. Respondents noted that career ladders supported retention efforts more than recruitment efforts, and were generally thought to be unrelated to engaging diverse candidates to fill vacancies. While health departments can develop comprehensive career ladders and succession planning programs, federal and state policies related to career and conditional employment and funding limitations for salary adjustments can impact consistent implementation across job classifications and position levels.^27^ The challenges of implementing comprehensive career ladder programs are compounded by civil service requirements, funding limitations, and complex recruitment processes that can inadvertently impact diverse candidates in attempts to mitigate bias.

This study also underscores the interconnectedness of career ladders and succession planning as strategies to maintain a skilled and diverse public health workforce. By providing opportunities for staff to develop leadership skills and offering additional educational benefits, the career ladder movement not only promotes internal advancement but also supports the continuity of operations and the retention of institutional knowledge during leadership changes. Nevertheless, these strategies are often made difficult given the challenges presented by state-level human resource policies that can curtail the effective implementation of career ladder and succession planning initiatives.

Despite the aging workforce, COVID-19 departures, and high turnover rates highlighting the immediate need for managing leadership transitions, few public health agencies engage in formal succession planning.^1-3,5,9^ These findings support the lack of use of succession plans by the health departments that participated in this study due in part to barriers identified. Darnell and Campbell (2015) found that while over 60% of local health departments were very or extremely concerned about recruiting and retaining qualified staff, only 40% were actively engaged in formal or informal succession planning. Public health agencies may encounter barriers preventing the use of succession planning including low prioritization, not having qualified mentors, or state-level policies that limit local health department flexibility in workforce decisions, which our findings also support.^2,5,9,28^

For governmental public health agencies, succession planning may help alleviate growing leadership vacancies due to retirement and high turnover rates.^2,3^ Similarly, succession planning may help improve workplace diversity by offering pathways to leadership for individuals who may have otherwise lacked the necessary education, training, and mentorship.^1,8,14^ Furthermore, succession planning may help improve operational efficiency in light of growing budgetary restrictions.^5^ Without addressing the barriers to implementing formal succession planning, these benefits will not be realized for governmental public health.

While this study provides a unique look at a newer approach to public health workforce recruitment and retention in health departments, limitations exist. Due to the nature of qualitative research, researcher bias was a potential limitation of this study. To account for such bias, a team-based analysis approach was used with checks for interrater agreement. Furthermore, as this study focused on health departments that had submitted a WDP as part of their accreditation materials for PHAB, these findings may be less reflective of public health practice within health departments not engaged in the accreditation process. Furthermore, due to the small sample size, results may not be representative to all health departments in the United States.

### Implications for policy and practice

- Career ladders are more useful for staff retention rather than recruitment.
- Positive implementation of career ladders is transparent and engages front-line staff.
- Career ladder implementation exists within the constraints of governmental public health, so while there are opportunities, there are also limitations, and it is important to be realistic.
- When developing career ladders, leaders should recognize that staff of all levels should have opportunities to increase their salaries and responsibilities or move into leadership roles if desired.
- Implementing career ladder strategies may be challenging due to historic promotional structures in job classifications. Existing approaches to step increases are often fixed within a defined role and have limited growth potential; however, career ladders can provide an opportunity for health department staff to refine their desired career trajectory over time. Health departments interested in implementing career ladders may consider best practices from the field that reflect both existing and potential structures focused on aligning higher levels with competencies that increase in skill complexity.
- Creating career ladders that promote institutional knowledge, on-the-ground training, and/or informal education will ensure opportunities for staff without specialized training.
- Career ladders that intersect across health department roles and functions may provide opportunities for staff who have reached the ceiling of a specific job path.
- While career ladders were not identified as a useful tool for recruitment, they could be. Practitioners should consider marketing the career ladders for recruitment.
- Successful career ladder implementation is linked to an organization-wide philosophy of staff development and may incorporate the importance of a positive work environment, workplace flexibility, mentoring, and strong leadership.

## Supplementary Material

qxae115_Supplementary_Data
